# Micromodification Mechanism and High-Temperature Rheological Properties of Activated Rubber/Styrene–Butadiene–Styrene Compound-Modified Asphalt

**DOI:** 10.3390/ma18112643

**Published:** 2025-06-04

**Authors:** Kai Zhang, Xuwen Zhong, Xukun Huang, Weihua Wan, Hai Zhou, Bin Liu

**Affiliations:** 1 Road Material and Structure Engineering Technology Research Center of Jiangxi Provincial, Jiangxi Communications Investment Maintenance Technology Group Co., Ltd., Nanchang 330200, China; kaizhang@whut.edu.cn (K.Z.); binliujky@126.com (B.L.); 2 School of Civil and Architectural Engineering, East China University of Technology, Nanchang 330013, China; 15007086333@163.com (X.Z.); charliez0112@163.com (H.Z.); 3 Dundee International Institute, Central-South University, Changsha 410075, China; shulkenh@csu.edu.cn; 4 Jiangxi Transportation Engineering Group Co., Ltd., Nanchang 330000, China

**Keywords:** conventional rubber, activated rubber, SBS modifier, modification mechanism, high-temperature rheological properties

## Abstract

Currently, research on the modification mechanisms of activated rubber/SBS (styrene–butadiene–styrene) composites and the microscopic processes involved remains limited. To investigate the impact of the rubber activation treatment combined with SBS modifier on asphalt modification, this study employs composite-modified asphalt formulations using either a conventional mix or activated rubber in conjunction with SBS. Infrared spectroscopy (IR) and scanning electron microscopy (SEM) were utilized to analyze the chemical components and microscopic morphology of the composite-modified asphalt following activation treatment. Microscopic analysis revealed that the asphalt stirred for 20 min has a characteristic peak with a wave number of 966 cm^−1^, while the characteristic peak with a wave number of 700 cm^−1^ is not obvious. That is, the asphalt sample contains the polybutadiene component and a reduced amount of the polystyrene component. Therefore, it can be inferred that the asphalt sample only contains activated rubber, along with less SBS modifier content. Traditional rubber undergoes significant expansion reactions during the mixing stage, but there are difficulties in degradation, which leave large particles and reduce the proportions of the lightweight asphalt components. However, active rubber and SBS mainly expand and degrade more completely during the shear stage, forming many micro-volume particles in asphalt. Additionally, frequency scanning and multiple creep recovery tests were conducted to evaluate the high-temperature rheological properties of the asphalt. The results indicate that activated rubber, doped at 20%, and SBS, doped at 2%, significantly enhance the high-temperature rheological properties of the composite-modified asphalt compared to base asphalt, exhibiting a 417.16% increase in the complex modulus at 64 °C and 1 Hz. Furthermore, these modifiers interact synergistically to improve modification efficiency.

## 1. Introduction

With the increasing amounts of waste rubber, recycling this material has become a problem to be solved, and at the same time, there is a certain need for high-performance asphalt in road construction projects. Waste rubber, as a modifier for road petroleum asphalt, can improve the performance of asphalt, and it involves recycling waste rubber, so rubber-modified asphalt has become a hot topic in the field of waste rubber recycling and road engineering [1,2]. Rubber-modified asphalt has better road performance and mechanical properties [3,4]. However, the molecular structure of conventional rubber is characterized by high cross-linking and poor compatibility with asphalt, which results in conventional rubber-modified asphalt being prone to a viscosity that is too high; additionally, the efficiency of the modified asphalt is low, and there are other problems [5,6,7]. These problems affect the application of rubber-modified asphalt in road engineering and hinder the recycling of waste rubber and the promotion of rubber-modified asphalt.

To solve the problems associated with the high viscosity and low modification efficiency of conventional rubber, some scholars advocate the use of conventional rubber through pretreatment, with the addition of other additives, etc. Pretreatment can change the internal chemical-bonding composition and crosslinking degree of conventional rubber materials to improve the road performance of conventional rubber asphalt [8,9,10]. Xiang et al. [11] pretreated conventional rubber using ultraviolet-light irradiation and found that after ultraviolet-light irradiation, the internal crosslinking degree and chemical-bonding changed, and the reaction with asphalt became more intense and improving the high-temperature shear resistance of the modified asphalt by 18–29%. Hosseinnezhad et al. [12] combined microwave and biological methods to activate conventional rubber, and the results showed that the breaking energy of activated rubber-modified asphalt is 135% of that of conventional rubber-modified asphalt. Feng et al. [13] conducted a study on the surface treatment of rubber powder with waste cooking oil. Edible oil surface treatment of rubber powder was investigated, and it was found that the solubility of treated rubber in asphalt increased, and the creep stiffness of the prepared modified asphalt was reduced by 9–34% compared to conventional rubber-modified asphalt.

Conventional rubber-modified asphalt also has poor compatibility with asphalt and poor high-temperature storage stability, and is prone to phase separation, leading to asphalt delamination and segregation [14,15,16]. Some scholars have reported that after microwave pretreatment, the specific surface area of conventional rubber increases, its compatibility with asphalt improves, and the high-temperature performance, fatigue resistance, and anti-aging properties of the activated rubber-modified asphalt are improved. Some scholars have found that with the pretreatment of conventional rubber with a macroscopic morphology and a fluffy flocculent structure, the rubber and asphalt contact area increases, and the asphalt’s compatibility is enhanced [17,18,19]. Mousavi et al. [20] added biomodifiers to reduce the degree of cross-linking within rubber and ground rubber adhered to finer powder to reduce the difference between the densities of the rubber and the asphalt to improve the stability of the high-temperature storage of asphalt, but this method is costly and difficult to generalize.

In addition, compound modification of asphalt by the combination of rubber with multiple modifiers can cause two modifiers to interact with each other and form a cross-linking network, which can improve the road performance of the asphalt and improve the defects of conventional rubber-modified asphalt to a certain extent [21,22,23,24]. Qian et al. [25] combined conventional rubber with SBS and reported that the addition of SBS can promote the degradation of conventional rubber in asphalt and improve the storage stability of modified asphalt at high temperatures. With the increase in SBS dosage at 60 °C and 0.1 kPa, the recovery rate of composite-modified asphalt can be increased by up to 20% compared to modified asphalt without SBS dosage. Kumar and Choudhary [26] combined conventional rubber with tire pyrolytic oil (TPO) and plastic pyrolytic oil to produce composite-modified asphalt with improved storage stability performance and rheological properties. The J_nr_ of modified bitumen at 60 °C increased by 63% and 56%, respectively. Furthermore, Rasool et al. [27] obtained desulfurized rubber by the twin-screw extrusion method and combined it with an SBS modifier to produce composite-modified asphalt with improved high-temperature performance. The desulfurization of the desulfurized rubber influenced its compatibility with SBS, thereby affecting the performance of the composite-modified bitumen.

Despite recent advancements, the micromodification mechanisms of conventional rubber/SBS and activated rubber/SBS composite-modified asphalts remain insufficiently studied. This paper investigates the micro-level modification mechanisms and the effects of rubber activation on composite-modified asphalt. In addition, the influence of modifier dosage on asphalt performance is examined, providing valuable insights for future research on rubber-SBS composite-modified asphalt.

## 2. Materials and Methods

### 2.1. Materials

#### 2.1.1. Base Asphalt

Modified asphalt is prepared by adding modifiers to base asphalt. Based on current domestic and international research on rubber-modified asphalt, this study conducted tests [28] on a modified base asphalt composed of 70# grade road petroleum asphalt. The softening point, penetration, elongation at 25 °C, and elongation at 5 °C were measured. The results are shown in Table 1.

#### 2.1.2. Activated Rubber

**Figure 1:**
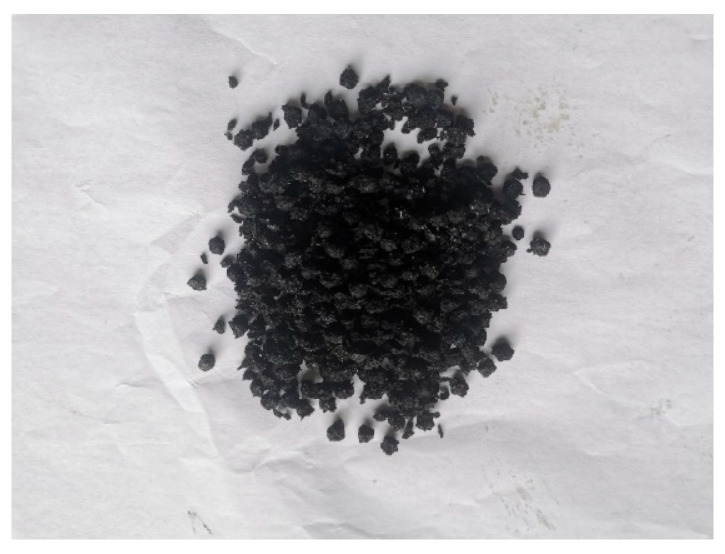
Activated rubber.

Activated rubber, which has higher surface activity than conventional rubber, can be obtained through surface pretreatment. In this study, activated rubber prepared using a twin-screw extruder after thermal-mechanical shear was selected for experimental investigation, based on relevant research in the field of road materials. The activated rubber is shown in Figure 1.

#### 2.1.3. Conventional Rubber

**Figure 2:**
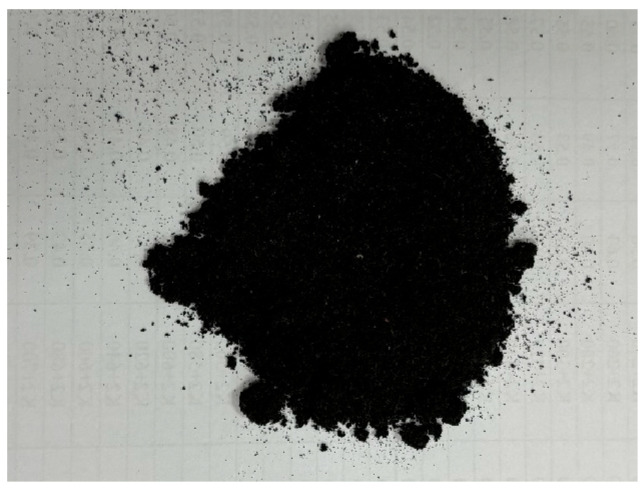
Conventional rubber.

Conventional rubber is primarily obtained from processed waste rubber. In this study, it is mainly used for comparison with activated rubber, with a mesh size of 40. The conventional rubber is shown in Figure 2.

#### 2.1.4. SBS Modifiers

**Figure 3:**
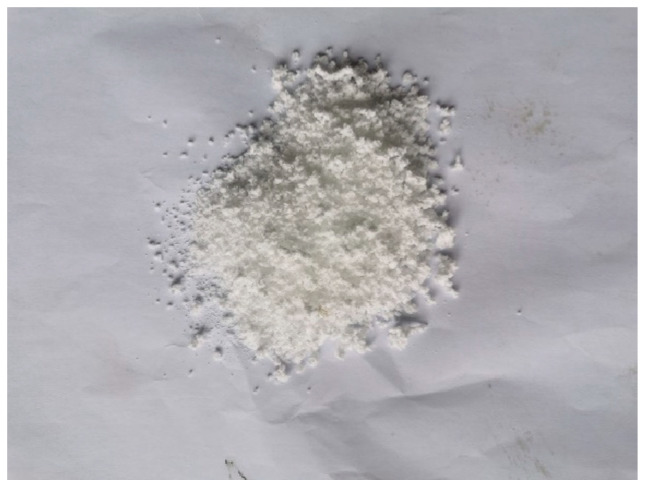
SBS modifier.

The SBS modifier is classified into star and linear types, and its structure influences its effect on asphalt modification. In current road construction projects, the linear SBS modifier is more commonly used. Therefore, this study uses the linear SBS modifier to prepare the modified asphalt. The SBS modifier is shown in Figure 3.

#### 2.1.5. Stabilizer

The activated rubber/SBS composite-modified asphalt system, which consists of two modifiers and asphalt, exists in a state of dynamic equilibrium. At high temperatures, mechanical mixing stops, and the two modifiers gradually precipitate from their uniformly dispersed state. This separation leads to variations in the vertical distribution of the components, ultimately reducing the overall performance of the rubber/SBS composite-modified asphalt.

Stabilizers used in road engineering generally have low molecular weights and specific functional groups. Modified asphalt stabilizers can be formulated to enhance high-temperature storage stability, heat resistance, and other properties of modified asphalt to a certain extent.

**Figure 4:**
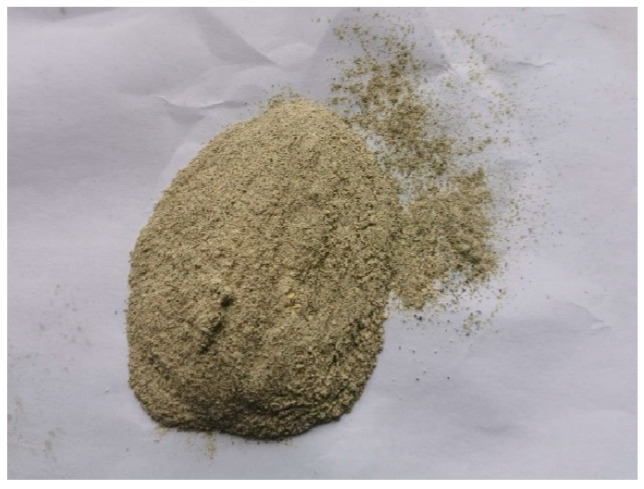
Sulfur.

Sulfur produced in industrial processes can be classified into three main types: refined sulfur, sublimated sulfur, and precipitated sulfur. In road construction projects, sublimated sulfur is commonly used as a stabilizer in the preparation of rubber-modified asphalt. Therefore, this study tests the use of sublimated sulfur as a stabilizer. Sulfur is shown in Figure 4.

#### 2.1.6. Composite-Modified Asphalt Preparation Process

**Figure 5:**
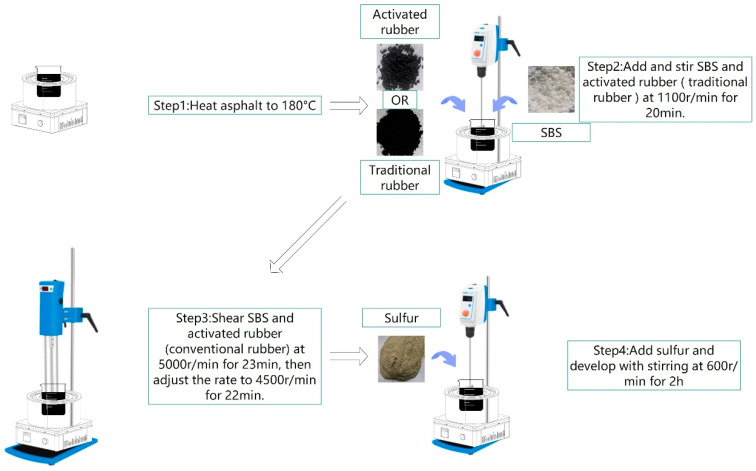
Preparation process.

For rubber-modified asphalt and desulfurized rubber-modified asphalt, the typical shear rate, temperature, and duration range from 2000 to 5000 r/min, 170–180 °C, and 45–60 min, respectively. The shear rate and duration for SBS-modified asphalt are similar, but the temperature is generally around 160 °C [29,30,31]. Considering the modification effect of both modifiers and the aging behavior of asphalt, this study adopts a higher shear rate and temperature with a shorter shear duration. The preparation process of the composite-modified asphalt is illustrated in Figure 5. The selected preparation temperature is 180 °C.

### 2.2. Methods

This study analyzes the micromechanism of two types of composite-modified asphalt, focusing on internal chemical composition, microstructure, and high-temperature rheological properties with varying modifier dosages [32]. The modification mechanism of rubber and SBS after activation treatment, as well as the influence of modifier dosage on the performance of the composite-modified asphalt, is also examined.

After material preparation, base asphalt, activated rubber/SBS composite-modified asphalt (in the preliminary miscible state), conventional rubber/SBS composite-modified asphalt, and activated rubber/SBS composite-modified asphalt were selected for infrared spectroscopy analysis. The tests aimed to analyze the chemical composition of the asphalt materials, with data collected in the mid-infrared range of 600–4000 cm^−1^.

Samples of conventional rubber/SBS and activated rubber/SBS composite-modified asphalt were taken at four stages of the preparation process: after 20 min of mixing, after 22 min of shearing, and after completion of development. Scanning Electron Microscopy (SEM) was used to examine the micromorphology of the asphalt at each stage, allowing analysis of the distribution of conventional rubber, activated rubber, and SBS within the asphalt, as well as the effects on asphalt microstructure.

The frequency sweep test is commonly used to evaluate the viscoelasticity and deformation resistance of asphalt. The test is conducted at temperatures ranging from 52 °C to 82 °C, with 6 °C intervals. A 25 mm plate is used, and the test frequency ranges from 0.1 to 100 Hz.

Multiple stress creep recovery (MSCR) tests were conducted at a temperature of 60 °C, with an initial conditioning creep stress of 0.1 kPa. The test included two loading stages: 0.1 kPa and 3.2 kPa, each applied over 10 continuous loading cycles. In each cycle, the sample was subjected to a constant stress for 1 s, followed by a 9 s recovery period. This test evaluates the elastic recovery capability of asphalt under two levels of shear creep stress.

The modifier dosages listed in Table 2 are based on the weight of the base asphalt.

## 3. Results and Discussion

### 3.1. Infrared Spectroscopy Analysis

#### 3.1.1. Base Asphalt

**Figure 6:**
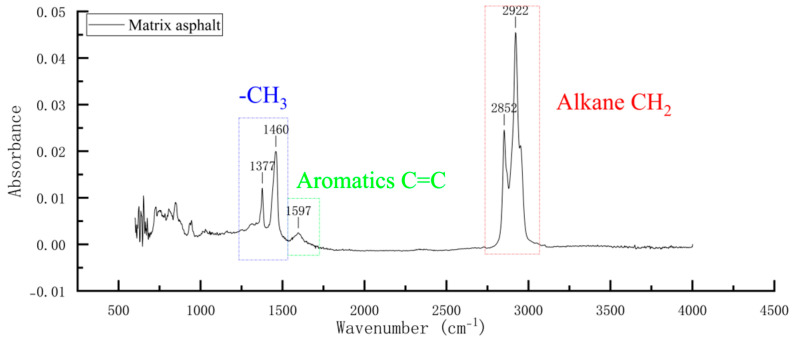
Infrared spectrum of base asphalt.

The infrared spectrum of the matrix asphalt is shown in Figure 6. Several prominent absorption peaks identified in the spectrum are listed in Table 3. Analysis of the results indicates that the 70# road petroleum asphalt used in this study contains saturated fraction, aromatic fraction, and aliphatic compounds.

#### 3.1.2. Activated Rubber/SBS Composite Modified Asphalt

**Figure 7:**
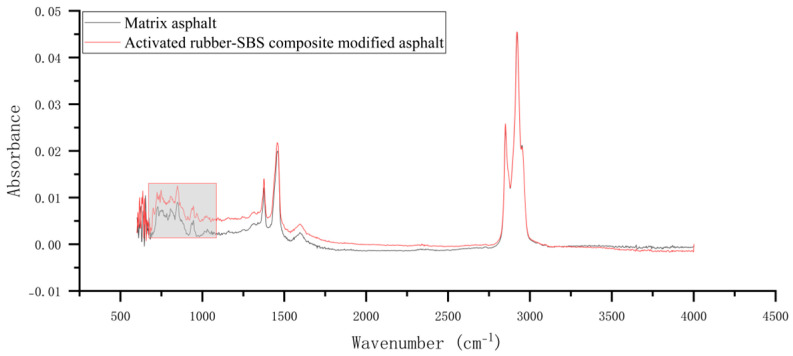
Infrared spectrum of activated rubber/SBS composite-modified asphalt.

**Figure 8:**
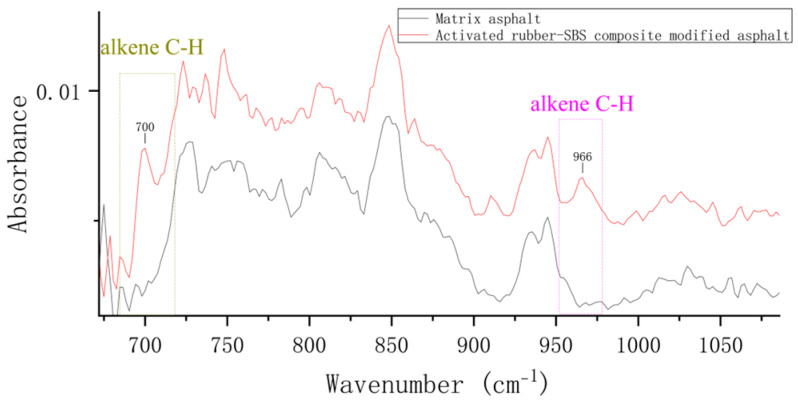
Locally magnified infrared spectrum of activated rubber/SBS composite-modified asphalt.

The infrared spectrum of the activated rubber/SBS composite-modified asphalt with 20% activated rubber and 2% SBS modifier is shown in Figure 7. By comparing the main peak features within the 600–1400 cm^−1^ wavenumber range, differences from the asphalt matrix spectrum can be observed. This region is locally enlarged to produce the infrared spectral map shown in Figure 8. The characteristic peaks of the activated rubber/SBS composite-modified asphalt are listed in Table 4.

According to the analysis results, compared with the base asphalt, the new components in the activated rubber/SBS composite-modified asphalt are mainly polybutadiene and polystyrene. The activated rubber contains polybutadiene, while the SBS modifier contains both polybutadiene and polystyrene. In addition, no new characteristic peaks were observed. This indicates that after adding activated rubber and SBS modifier to the base asphalt, no significant new chemical compounds are formed, suggesting that the interaction between the activated rubber, SBS modifier, and asphalt is primarily a physical swelling reaction.

**Figure 9:**
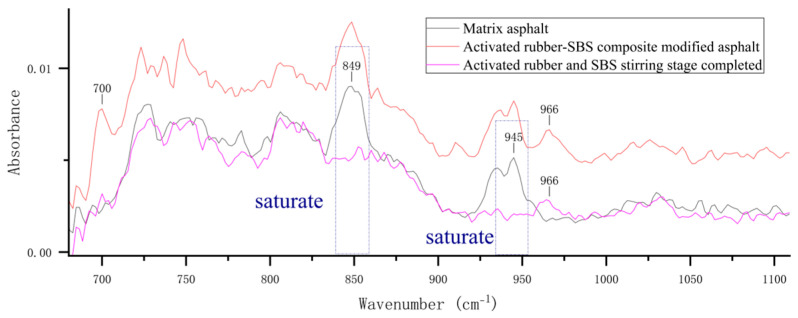
Infrared spectrum of activated rubber/SBS composite-modified asphalt after completion of the stirring stage.

To analyze the compositional changes in activated rubber/SBS composite-modified asphalt during preparation, this study compares the infrared spectrum of the asphalt in its preliminary mixed state after mixing and stirring for 20 min with that of asphalt containing activated rubber and SBS modifier. A locally magnified map is shown in Figure 9.

According to the locally magnified figure, after stirring the asphalt for 20 min, a characteristic peak at the wavenumber 966 cm^−1^ was observed, while the peak at 700 cm^−1^ was not prominent. This indicates a lower presence of polybutadiene and polystyrene components in the asphalt samples. It can be inferred that the asphalt samples contained smaller amounts of activated rubber and SBS modifier. The reason for this difference is likely that the activated rubber and SBS modifiers were not sheared, resulting in larger particle sizes. Consequently, the infrared spectroscopy signal from the asphalt samples was weaker, and the modifiers were unevenly distributed in the sample. However, new characteristic peaks appeared in the infrared spectra after 20 min of stirring, indicating that the modifiers begin to dissolve during the initial mixing stage of asphalt preparation.

At the same time, after stirring the asphalt for 20 min, the two characteristic peaks at 849 cm^−1^ and 945 cm^−1^ did not appear, while the two absorption peaks of the activated rubber/SBS composite-modified asphalt became apparent. These two characteristic peaks represent the saturated fraction of the asphalt. The reason is that during the mixing stage, part of the SBS and activated rubber begin to absorb the light components of the asphalt and swell. At this stage, some SBS and activated rubber have started absorbing the light components to dissolve, but have not yet reached the degradation stage, which leads to a decrease in the saturated content of the asphalt. After the development process is completed, the activated rubber and SBS cause the degradation of a significant amount of asphalt, releasing the absorbed saturated components back into the asphalt, which results in an increase in the saturated content.

#### 3.1.3. Conventional Rubber/SBS Composite-Modified Asphalt

**Figure 10:**
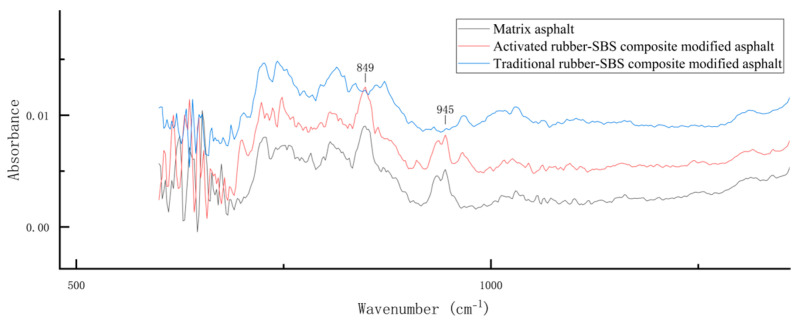
Locally magnified infrared spectrum of conventional rubber/SBS composite-modified asphalt.

The locally magnified spectrum of conventional rubber/SBS composite-modified asphalt, prepared with 20% conventional rubber and 2% SBS modifier, is shown in Figure 10. The results indicate that this asphalt lacks the two characteristic peaks at 849 cm^−1^ and 945 cm^−1^, suggesting that its saturated content is lower than that of the base asphalt and the activated rubber/SBS composite-modified asphalt. In asphalt, conventional rubber absorbs the saturated fraction and expands in volume. However, its dissolution efficiency is low, and it cannot re-release the absorbed saturated components effectively. As a result, the saturated fraction in conventional rubber/SBS composite-modified asphalt is reduced.

**Figure 11:**
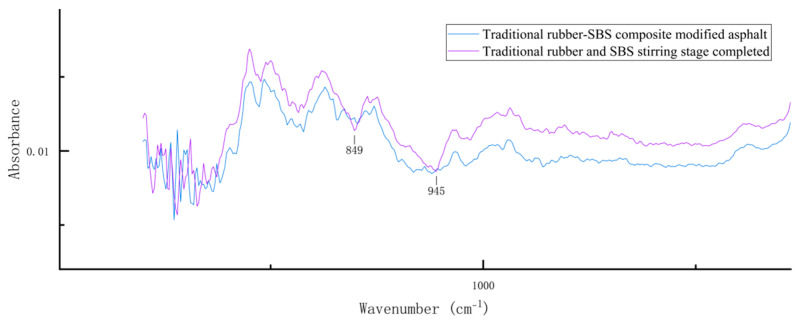
Locally magnified infrared spectrum of conventional rubber/SBS composite-modified asphalt after completion of the stirring stage.

To analyze the compositional changes in conventional rubber/SBS composite-modified asphalt during the preparation process, this study uses infrared spectra of asphalt samples mixed with the two modifiers for 20 min. The results are shown in Figure 11.

The two negative peaks at 849 cm^−1^ and 945 cm^−1^ in the spectrum of the conventional rubber/SBS composite asphalt, stabilized for 20 min, indicate that the saturated fraction of the composite asphalt is complete in the mixing stage of preparation, as compared to the decrease in the content of the saturated fraction. These results indicate that the conventional rubber/SBS composite asphalt mixing process can lead to more pronounced dissolution reactions. From these results, it can be inferred that during the mixing process, the conventional rubber/SBS composite-modified asphalt undergoes a more pronounced swelling reaction. This occurs when the conventional rubber absorbs a large number of light components, causing volume expansion, but before it enters the degradation stage. As a result, a significant amount of saturated content in the asphalt is absorbed, leading to a reduction in its content. After asphalt development, partial degradation of the conventional rubber occurred, and some of the saturated fraction was released, leading to an increase in the asphalt’s saturated fraction. However, the overall content remained lower than that of the matrix asphalt and activated rubber/SBS composite-modified asphalt, which further indicates that the degradation efficiency of conventional rubber in asphalt is low.

Based on the initial miscible state of the activated rubber/SBS and conventional rubber/SBS composite-modified asphalts, the infrared spectra show that the solvation reactions of the activated rubber and SBS modifier primarily occur during the shear and development stages. In contrast, conventional rubber undergoes a strong solvation reaction during the mixing stage. This is mainly because the activated rubber and SBS modifier, before shear, are in the form of larger granular materials with a low contact area with the asphalt, resulting in a lower swelling reaction efficiency. After shearing, the two modifiers decrease in volume, significantly increasing their contact area with the asphalt. At this stage, the swelling of the activated rubber/SBS composite-modified asphalt intensifies, and the particle size of the conventional rubber is much smaller than that of the unsheared activated rubber and SBS modifier. Therefore, during the mixing stage, the modifier can absorb a large amount of lightweight components due to the more intense swelling reaction.

However, due to the high internal crosslinking of the conventional rubber, the efficiency of the degradation reaction after the volume expansion is low. As a result, part of the absorbed saturated fraction cannot be released, leading to a decrease in the saturated fraction of the conventional rubber/SBS composite-modified asphalt. Rubber activation breaks the internal S-S bonds and interrupts the C-S bonds, improving compatibility with the asphalt and thereby increasing the efficiency of the degradation reaction. During the swelling reaction, a large number of lightweight components are absorbed, which are then released into the asphalt during the degradation phase. Due to its material properties and high compatibility with asphalt, SBS can undergo a more efficient reaction in asphalt, leading to a higher degree of degradation and interaction with the asphalt. Therefore, compared to the base asphalt, the content of lightweight components in the activated rubber/SBS composite-modified asphalt is not significantly reduced, while it is significantly reduced in the conventional rubber/SBS composite-modified asphalt.

### 3.2. SEM Scanning Analysis

**Figure 12:**
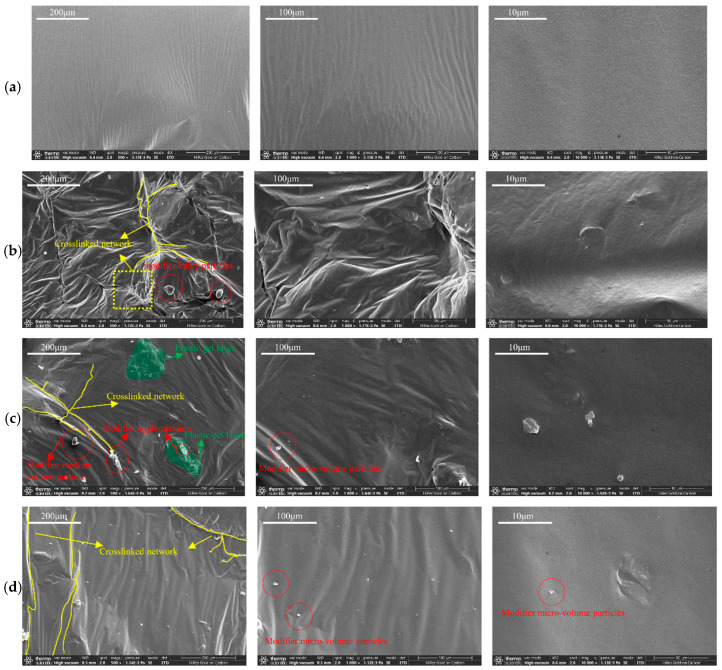
SEM images of activated rubber/SBS composite modified asphalt at different reaction stages: (**a**) complete stirring, (**b**) shearing for 22 min, (**c**) complete shearing, and (**d**) completed development.

**Figure 13:**
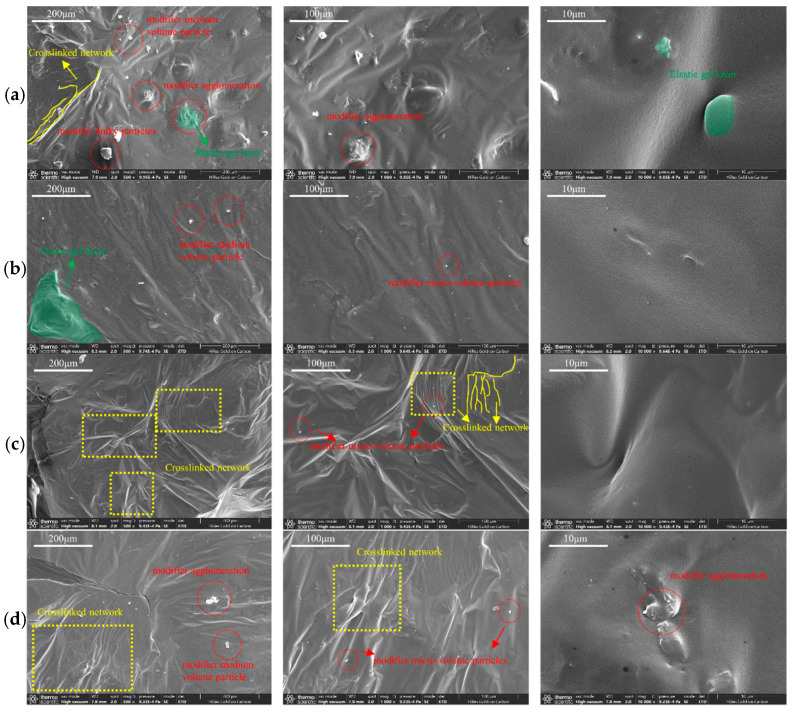
SEM images of conventional rubber/SBS composite modified asphalt at different reaction stages: (**a**) complete stirring, (**b**) shearing for 22 min, (**c**) complete shearing, and (**d**) completed development.

The microscopic morphology of activated rubber/SBS composite-modified asphalt with 20% rubber and 2% SBS modifier, as well as that of conventional rubber/SBS composite-modified asphalt at different preparation stages, was observed using SEM, as shown in Figure 12 and Figure 13.

#### 3.2.1. Activated Rubber/SBS Composite Modified Asphalt

Figure 12 shows the microscopic morphology of activated rubber/SBS composite-modified asphalt at different stages of the preparation process. Figure 12a shows that after mixing, the asphalt surface is relatively flat, and no modifier particles are visible, indicating a low degree of dissolution of the activated rubber and SBS modifier in the asphalt. At this stage, the modification effect on the asphalt is weak. This is because the activated rubber and SBS modifier have large particle sizes, and in the absence of shear, their reaction efficiency with asphalt is low.

After 22 min of shearing, as shown in Figure 12b, the microscopic morphology of the activated rubber/SBS composite-modified asphalt changed. A large number of bumps and depressions appeared on the surface, and activated rubber and SBS modifier particles began to emerge. The decrease in the volume of the activated rubber and SBS modifier after the reduction in shear particle size is due to the absorption of a large amount of asphalt from the light components during the swelling reaction, which leads to the formation of an elastic gel layer on the surface. At this point, the volume of the activated rubber and SBS modifier particles increased, and the relative distance between the modifier particles decreased significantly, gradually forming a cross-linked structure in the asphalt. This resulted in a change in the microscopic morphology of the asphalt surface.

Figure 12c shows the microscopic morphology of the activated rubber/SBS composite-modified asphalt after shear. The microscopic morphology of the asphalt surface gradually flattens, with a reduction in the volume of modifier particles and a decrease in bumps and depressions. At the same time, modifier agglomeration occurs, leading to the formation of a small number of micro-sized modifier particles. The reason is that, after shear, a large number of activated rubber and SBS modifier particles with reduced volume undergo an asphalt swelling reaction. This causes a significant increase in the volume of modifier particles due to swelling, leading to the formation of a viscous elastic gel layer. As a result, some of the larger modifier particles adhere to each other, forming agglomerates. At the same time, some of the modifier particles have transitioned from the asphalt swelling stage to the degradation stage, resulting in a reduction in the volume of large modifier particles and their splitting into micro-sized particles. At this point, the volume of asphalt modifier particles on the surface decreases, and the elastic gel layer reduces the impact of the asphalt surface morphology, leading to an improvement in flatness.

Figure 12d shows the development of activated rubber/SBS composite-modified asphalt, where the surface micromorphology further improved in flatness. The larger modifier particles disappeared, and a large number of micro-sized modifier particles appeared. The reason is that, after the development stage, the activated rubber and SBS modifier particles in the composite-modified asphalt fully dissolved and degraded. The large modifier particles split into many micro-sized particles, and the elastic gel layer formed on the surface further reduced the volume of modifier particles, improving the asphalt’s surface morphology and flatness.

In the shear stage, a large volume increase due to swelling, along with the formation of elastic gel layers, significantly alters the surface micromorphology of the asphalt. In the second half of the swelling stage, many swollen modifier particles transition into the degradation stage. At this point, the elastic gel layer in the asphalt helps reduce the impact on the surface micromorphology, allowing the flatness of the asphalt to begin restoring. Also, a large number of sufficiently swollen modifier particles begin to degrade and break into many micro-sized particles that are dispersed in the asphalt, causing the asphalt’s surface micromorphology to improve further. The main phase of the degradation reaction is the development stage. At this point, a large number of sufficiently swollen modifier particles begin to degrade and break into many micro-sized particles dispersed in the asphalt. The elastic gel layer in the asphalt essentially disappears, and the surface micromorphology of the asphalt continues to improve, with further enhancement of its flatness.

#### 3.2.2. Conventional Rubber/SBS Composite Modified Asphalt

Figure 13 shows the microscopic morphology of conventional rubber/SBS composite-modified asphalt at different stages of the preparation process. Figure 13a shows that after the completion of the mixing stage, the asphalt’s microscopic morphology formed an uneven structure, with a large number of large- and medium-sized modifier particles and signs of modifier agglomeration. This is due to part of the conventional rubber in the asphalt absorbing lightweight components during the dissolution reaction.

Figure 13b,c show that during the shear stage and after shearing, only minor changes in the microscopic morphology and flatness of the asphalt are observed. This indicates that the conventional rubber has not undergone significant degradation and that the asphalt is still in a swollen state with an increase in volume. At the same time, a small number of small-volume modifier particles are observed on the asphalt surface, likely resulting from the SBS modifier beginning to dissolve and degrade within the asphalt, splitting into micro-sized particles.

Figure 13d shows the microscopic morphology of the conventional rubber/SBS composite-modified asphalt after the completion of the process. Compared to the asphalt surface before the process, the microstructure of the asphalt surface after completion is still largely influenced by the volume of modifier particles, which are presumed to remain undegraded. Additionally, the microscopic morphology of the asphalt surface during the shear stage shows no significant changes.

Overall, the small particle size of conventional rubber allows for extensive dissolution during the mixing stage, leading to initial modification of the asphalt as the volume of the conventional rubber expands. However, as the preparation process progresses, the large volume of expanded conventional rubber becomes difficult to dissolve in the asphalt, resulting in the presence of many large- and medium-sized particles in the conventional rubber/SBS composite-modified asphalt at various stages of preparation. The larger particles primarily consist of an undegraded conventional rubber core surrounded by a gel layer. These particles are connected by friction to form a high-viscosity crosslinked structure, significantly increasing the viscosity of the asphalt. At the same time, the larger particles are more prone to segregation, which reduces the asphalt’s high-temperature storage stability.

Activated rubber has a large particle size and requires shearing to react with asphalt in significant amounts. After activation treatment, the S-S bond and C-S bonds in conventional rubber are broken, improving its compatibility with asphalt. This leads to swelling and volume increases in the asphalt, after which the material gradually degrades and is evenly distributed in the asphalt. Therefore, after the development of the activated rubber/SBS composite-modified asphalt, the disappearance of large-volume particles left only a large number of micro-sized particles. The effect of the SBS modifier on the asphalt reaction process is similar to its effect on the activation of rubber in the asphalt. The swelling reaction leads to gradual degradation and uniform dispersion of the activated rubber in the asphalt, making it a more effective composite modifier. This improves the asphalt’s performance while also reducing its viscosity.

### 3.3. Analysis of High-Temperature Rheological Properties of Asphalt

#### 3.3.1. Frequency Scanning

According to the principle of time-temperature equivalence, the complex modulus curve of the activated rubber/SBS composite-modified asphalt obtained under different temperature conditions can be shifted along the logarithmic frequency axis using a displacement factor. This allows the complex modulus under various temperatures to be superimposed into a single, smooth curve, known as the master curve of the complex modulus. This master curve can be used to predict the rheological properties of composite-modified asphalt across a wide range of temperatures and frequencies. This test was conducted according to AASHTO Designation: T 315 (2022), with test temperatures of 52, 58, 64, 70, 76, and 82 °C, and a test frequency range of 0.1–100 Hz.

The displacement factor for the complex modulus curve of activated rubber/SBS composite-modified asphalt at different test temperatures can be calculated using the WLF formula, as shown in Equation (1).

(1) $$\mathrm{lg}\alpha_{T}=\frac{-C_{1}\left(T-T_{0}\right)}{C_{2}+\left(T-T_{0}\right)}$$

$\mathrm{lg}\alpha_{T}$—displacement factor;

$C_{1}$,$C_{2}$—constant values;

T—test temperature (°C);

$T_{0}$—reference temperature (°C).

In this paper, a sigmoidal model is used to fit the translated complex modulus. The sigmoidal model is shown in Equation (2).

(2) $$\mathrm{lg}\left(G^{*}\right)=\delta +\frac{\alpha }{1+e^{\beta +\gamma \left(\mathrm{lg}f_{r}\right)}}$$

$G^{*}$—complex modulus (MPa);

$f_{r}$—curtailment frequency (Hz);

δ—low-frequency asymptote of lgG*;

δ+α—high-frequency asymptotic values of lgG*;

β, γ—shape adjustment parameters.

**Figure 14:**
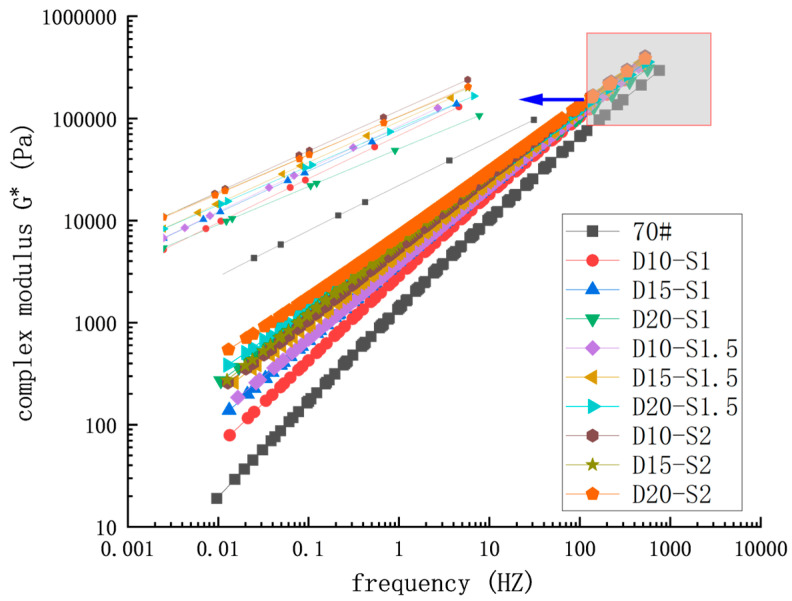
Complex modulus main curve.

The reference temperature selected for the complex modulus master curve in this paper is 64 °C. Figure 14 shows the master curve of the complex modulus for each asphalt sample, obtained by translational fitting of the test data.

As the modifier dosage increases, the complex modulus of the composite-modified asphalt improves further. At a frequency of 1 Hz, when the activated rubber dosage in the composite-modified asphalt increases from 10% to 20%, the complex modulus improves by 85.3%. Similarly, when the SBS dosage increases from 1% to 2%, the complex modulus improves by 89.0%. When the activated rubber dosage increases from 10% to 20% and the SBS dosage increases from 10% to 20%, or when the activated rubber dosage increases from 10% to 20% and the SBS dosage increases from 1% to 2%, the complex modulus increases by 150.6%. Increasing the dosage of activated rubber or SBS in asphalt leads to a greater amount of these modifiers participating in the swelling reaction, enhancing the asphalt’s modification. At the same time, the dispersion of micro-sized asphalt particles increases as the crosslinking network density grows, thereby improving the composite-modified asphalt’s resistance to shear deformation.

#### 3.3.2. Multiple Stress Creep Recovery Test

The multiple Stress creep recovery test is a commonly used method for evaluating the high-temperature rheological properties of modified asphalt, and it more effectively simulates the loading conditions that asphalt pavements experience during actual use. This test was conducted according to AASHTO Designation: T 350–19 [33], with a test temperature of 60 °C in this paper.

**Figure 15:**
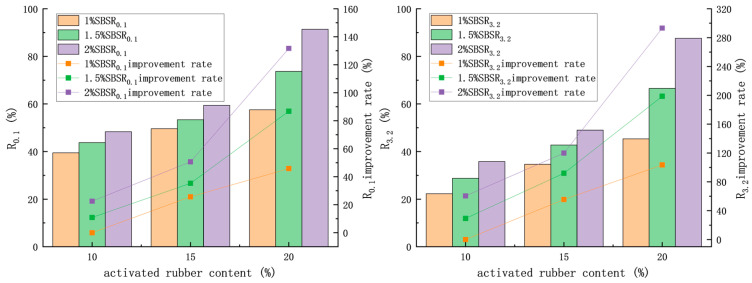
Asphalt recovery rate under stress.

**Figure 16:**
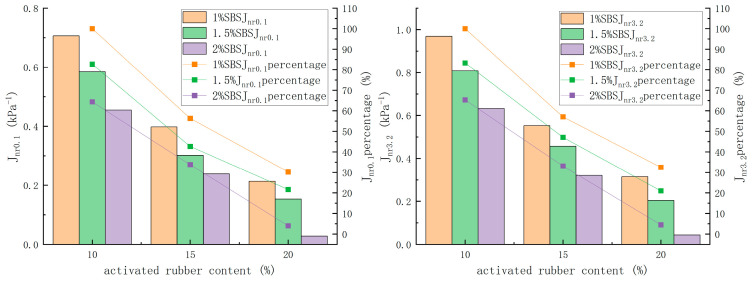
Nonrecoverable creep compliance under stress.

The test results are presented in Figure 15 and Figure 16. The recovery rates at stresses of 0.1 kPa and 3.2 kPa are calculated using Equations (3) and (4):

(3) $$R_{0.1}=\frac{SUM\left[\in_{r}\left(0.1,N\right)\right]}{10}\;\mathrm{for}\;N\;=\;11\;\mathrm{to}\;20$$

(4) $$R_{3.2}=\frac{SUM\left[\in_{r}\left(3.2,N\right)\right]}{10}\;\mathrm{for}\;N\;=\;1\;\mathrm{to}\;10$$

$R_{0.1}$—recovery percent at 0.1 kPa;

$R_{3.2}$—recovery percent at 3.2 kPa;

$\in_{r}$—recovery rate under a single cycle.

The nonrecoverable creep compliance at stresses of 0.1 kPa and 3.2 kPa is calculated using Equations (5) and (6):

(5) $$J_{\mathrm{nr}0.1}=\frac{SUM\left[J_{nr}\left(0.1,N\right)\right]}{10}\;\mathrm{for}\;N\;=\;11\;\mathrm{to}\;20$$

(6) $$J_{\mathrm{nr}3.2}=\frac{SUM\left[J_{nr}\left(3.2,N\right)\right]}{10}\;\mathrm{for}\;N\;=\;1\;\mathrm{to}\;10$$

$J_{nr0.1}$—nonrecoverable creep compliance at 0.1 kPa;

$J_{nr3.2}$—nonrecoverable creep compliance at 3.2 kPa;

$J_{nr}$—nonrecoverable creep compliance under a single cycle.

With an activated rubber dosage of 10% and an SBS dosage of 1%, the recovery rate of compound-modified asphalt increased by 18.1% and 23.1% under 0.1 kPa and 3.2 kPa conditions, respectively, when the activated rubber dosage was increased from 10% to 20%. Meanwhile, the nonrecoverable creep compliance decreased by 69.8% and 67.5%, respectively. When the SBS dosage was increased from 1% to 2%, the recovery rate increased by 8.9% and 13.5%, respectively, and the nonrecoverable creep compliance decreased by 35.7% and 34.7%, respectively. From the results, it can be inferred that the SBS micro-sized particles, formed by the crosslinking network structure, enhance the composite-modified asphalt’s ability to recover from load stress. At the same time, the deformation of the rubber in composite-modified asphalt has a more significant impact on the recovery ability than the SBS. This is because the high elasticity of the activated rubber, along with the characteristics of the composite-modified asphalt, enhances the overall elasticity of the material.

When the activated rubber dosage increased from 10% to 20% and the SBS modifier was raised to 2%, the recovery rate of the composite-modified asphalt at 0.1 kPa and 3.2 kPa increased by 52.0% and 65.3%, respectively, while the nonrecoverable creep compliance decreased by 96.0% and 95.5%, respectively. Simultaneously increasing both modifier dosages improves the deformation recovery ability of composite-modified asphalt more than increasing a single modifier dosage, indicating that the higher dosage of one modifier positively impacts the efficiency of the other modifier’s effect. This may be due to the activation of both rubber and SBS in the asphalt, which enhances the efficiency of the modified composite asphalt and the formation of a crosslinking network structure between the rubber and SBS. Compared to the crosslinked network structure of a single modifier, the network structure of the modified asphalt enhances its performance.

## 4. Conclusions

### 4.1. Conclusions

In this paper, changes in the composition and micro-morphology of activated rubber-SBS composite-modified asphalt during the modification process were analyzed using infrared spectroscopy and SEM scanning. The impact of rubber activation on the modification process of composite-modified asphalt was also discussed. The effects of activated rubber and SBS dosage on the properties of composite-modified asphalt were also investigated using frequency scanning and MSCR tests:

(1) According to the infrared spectroscopy results, conventional rubber in the asphalt dissolution reaction absorbs a large number of light components, which are released during the degradation process. However, the high degree of internal crosslinking in conventional rubber results in low degradation efficiency, causing the light component content of conventional rubber/SBS composite-modified asphalt to be lower than that of the base asphalt;

(2) The swelling reaction process of activated rubber and SBS modifier in asphalt is similar to that of conventional rubber, but the former two are more compatible with asphalt and degrade more easily. After the swelling reaction, no new chemical compositions are formed in the composite-modified asphalt, indicating that the swelling reaction of activated rubber and SBS modifier in the asphalt is primarily physical;

(3) Conventional rubber undergoes a swelling reaction during the mixing stage. When the volume of conventional rubber expands, the initial modification of the asphalt occurs. However, due to the high degree of cross-linking within the rubber, many large-volume particles of conventional rubber remain in the asphalt even after the modification process. These particles negatively affect both the performance and viscosity of the composite-modified asphalt;

(4) The two types of modifiers have high compatibility with asphalt, and after shear, they undergo significant dissolution reactions. In the development stage, complete degradation occurs, and a large number of microvolume particles are dispersed in the asphalt. These microvolume particles can form a cross-linked network structure, enhancing the performance of the asphalt;

(5) Activated rubber and SBS improve the asphalt’s resistance to shear deformation under high-temperature conditions and enhance its deformation recovery ability. Increasing the dosage of both modifiers further improves the asphalt’s high-temperature resistance and enhances its deformation performance. The interaction between the two types of modifiers enhances the modification efficiency of asphalt’s resistance to high-temperature deformation.

### 4.2. Limitations and Future Work

This research primarily focuses on the microscopic aspects of rubber-modified asphalt, with limited exploration of the processing technology of activated rubber and the fatigue resistance of activated rubber-modified asphalt. However, due to time and resource constraints, a comprehensive analysis of related research is lacking. Additionally, there has been no in-depth exploration of the road performance, durability, and other aspects of asphalt mixtures. Future research will comprehensively address the topics mentioned above.

## Figures and Tables

**Table 1:** Basic performance of base asphalt.

Pilot Projects	Unit	Test Values
Penetration (25 °C)	0.1 mm	71.6
Softening point	°C	49.6
Ductility (25 °C)	cm	>100
Ductility (5 °C)	cm	5.6

**Table 2:** Modifier dosage.

Sample Number	Dosage of Activated Rubber (%)	Dosage of SBS (%)
D10-S1	10	1.0
D15-S1	15	1.0
D20-S1	20	1.0
D10-S1.5	10	1.5
D15-S1.5	15	1.5
D20-S1.5	20	1.5
D10-S2	10	2.0
D15-S2	15	2.0
D20-S2	20	2.0

**Table 3:** Base asphalt absorption peaks.

Wavenumber (cm^−1^)	The Peak of a Telescope	Characterizing Component
2922	Alkane CH_2_ antisymmetric stretching peaks	saturate
2852	Alkane CH_2_ symmetric stretching peaks	saturate
1597	Aromatic C=C stretching peaks	aromatic
1460	Aliphatic CH3 asymmetric variant angles	aliphatic compound
1377	Aliphatic CH_3_ symmetry variant	aliphatic compound

**Table 4:** Absorption peaks of activated rubber/SBS composite-modified asphalt.

Wavenumber (cm^−1^)	The Peak of a Telescope	Characterizing Component
966	trans-olefin CH peak	polybutadiene
700	cis-olefin CH peak	polystyrene

## Data Availability

The data that support the findings of this study are available from the corresponding author upon reasonable request.
